# REMP: A unique dataset of rare and endangered medicinal plants in Bangladesh for sustainable healing and biodiversity conservation

**DOI:** 10.1016/j.dib.2024.110895

**Published:** 2024-09-03

**Authors:** Mohammad Manzurul Islam, Sanjida Rahman, Nahida Hoque, Md. Al Mamun, Md. Sultan Moheuddin, Md. Sawkat Ali, Mohammad Rifat Ahmmad Rashid, Saleh Masum, Md. Hasanul Ferdaus, Nishat Tasnim Niloy, Md. Atiqur Rahman

**Affiliations:** a Department of Computer Science and Engineering, East West University, Aftabnagar, Dhaka, Bangladesh; b Department of Information and Communication Engineering, University of Rajshahi, Bangladesh

**Keywords:** Medicinal plants, Rare plants, Endangered plants, Threatened plants, Machine learning

## Abstract

In Bangladesh, there are significant number of medicinal plants, but currently no comprehensive record of these valuable species is publicly available. Alarmingly, some of these plants are in a precarious state of endangerment. Therefore, we are creating a unique dataset of Bangladeshʼs rare, endangered, and threatened medicinal plants to support conservation efforts. It will help us to track and conserve endangered plant species, ensuring a more organized approach to research and preservation efforts. We conducted on-site visits to the National Botanical Garden and The Government Unani and Ayurvedic Medical College, capturing photographs of these plants in optimal sunlight conditions at various times of the day. This involved fieldwork, detailed image annotations, dataset organization, diversity augmentation, and contribution to the preservation of our natural heritage. We have collected a total of 16 types of rare and endangered medicinal plant leaf photos to create our unique dataset consisting of a total of 3494 images. This dataset will help researchers in biodiversity conservation through building efficient machine learning models and applying advanced machine learning techniques to identify rare and endangered medicinal plants.

Specification Table

**Table:** 

Subject	Computer Sciences, Agriculture Sciences, Pharmaceutical Sciences.
Specific subject area	Computer Vision, Image Processing, Image Classification, Machine Learning.
Type of data	The images are in JPG format (total 3494 jpg files) having 640 × 480 pixel dimensions and 96 × 96 dpi image resolution.
Data collection	This dataset comprises a total of 3494 original leaf images, which have been categorized into 16 different plant leaf classes. The images were captured using (i) Samsung Galaxy S21+ 5 G, and (ii) Redmi Note 11 smartphone cameras.
Data source location	The data were collected from the following locations: 1. The National Botanical Garden of Bangladesh, Mirpur, Dhaka - 1216. (Latitude: 24° 00′ 0.00″ N, Longitude: 90° 00′ 0.00″ E) 2. The Government Unani and Ayurvedic Medical College and Hospital, Mirpur Bridge Road, Dhaka– 1216. (Latitude: 23° 80′ 43.99″ N, Longitude: 90° 37′ 83.48″ E.)
Data accessibility	Repository name: Mendeley Data Data identification number: 10.17632/hnwrxg8zm8.1 Direct URL to data: https://data.mendeley.com/datasets/hnwrxg8zm8/1

## 1 Value of the Data

• The dataset comprises 16 different types of medicinal plant leaves among which (i) six types are rare, (ii) four types are endangered, and (iii) six types are threatened medicinal plant leaves collected from different regions of Dhaka, Bangladesh.
• Conservationists can utilize the dataset to monitor and protect endangered or threatened medicinal plant species. Identifying these species through leaf images is crucial for their preservation.
• The dataset could be a valuable resource for researchers to identify rare and endangered medicinal plants using machine learning, deep learning, or other artificial intelligence methods. This information could also be beneficial for botanists, pharmacists, and others interested in working with medicinal plants.
• The dataset is valuable for the educational sector as it can help to identify medicinal plants and to know about their significance.

## 2 Background

Medicinal plants have been used for their therapeutic properties for centuries in various traditional systems of medicine around the world. Anti-inflammatory, antiviral, antitumor, antimalarial, and analgesic properties are only some of the therapeutic benefits that medicinal plants have been associated with [2]. Medicinal plants are used for both modern and traditional medicine. Some plants are used directly as primary medicinal treatment, and some are used to make medicine. More than 50,000 plants are used for medicinal purposes by people worldwide [4]. According to the U.S. Forest Service, 40 % of pharmaceuticals in the Western world are made up of plants [4]. Medicinal plants are crucial for humans and other animals, too. The main purpose of this dataset is to help conservationists monitor, protect, and preserve these important medicinal plant species by identifying them effectively. The researchers can utilize this dataset by training and evaluating machine learning, deep learning, and transfer learning-based models for the identification of undetected such medicinal plants in the wild. This dataset could be an asset for pharmacists, researchers, botanists, and conservationists.

In this work, we aim to create a dataset of rare, endangered and threatened medicinal plants of Bangladesh. We collected leaves from only healthy plants, ensuring no harm to their survival. We found 16 types of such plants which are not available in existing public datasets. Borkatulla et al. [4] and Islam et al. [31] collected 10 classes of plant images, however, only one plant class from each of these datasets are common with our dataset. The work in [31] did not consider cleaning the noise from the dataset which may impact machine learning algorithms. A recent work by Pushpa et al. [32] compiled an Indian plant-based dataset having 40 different types of plants. Many of these plants from this dataset include regular vegetable (e.g., onion, spinach), fruits (e.g., pomegranate, mango), flower (e.g., rose, jasmine) plants that could be alternatively used as medicinal plants. However, similar to [4,31], this work also does not address the rare, endangered and threatened medicinal plants as focused in our dataset. To the best of our knowledge, our dataset is unique as it only collected the rare, endangered and threated medicinal plants in Bangladesh. Table 1 compares our dataset with existing medicinal plant-based datasets.

**Table 1:** Comparison with existing datasets.

SL	Plant / Classes	Number of images			
		Our Dataset	Borkatullaa et al. [4]	Islam et al. [31]	Pushpa et al. [32]
*1*	*Alternanthera brasiliana* L. *kuntze*	201	X	X	X
*2*	*Hemidesmus indicus (Linn.) R. Br.*	158	X	X	X
*3*	*Achyranthes aspera*	280	X	X	X
*4*	*Ayapana triplinervis*	294	X	X	X
*5*	*Gloriosa superba*	138	X	X	X
*6*	*Andrographis paniculata wall. ex nees*	294	X	X	90
*7*	*Datura tramonium* L.	276	X	X	X
*8*	*Gynura procumbens (Lour.) merr.*	238	500	X	X
*9*	*Artocarpus chama*	238	X	X	X
*10*	*Eleutherine bulbosa*	194	X	X	X
*11*	*Boerhavia diffusa* L.	407	X	X	X
*12*	*Clerodendrum indicum* L. *kuntze*	134	X	X	X
*13*	*Justicia adhatoda* L.	151	X	200	X
*14*	*Ocimum gatissimum* L.	154	X	X	X
*15*	*Asparagus racemosus willd*	108	X	X	X
*16*	*Rauwolfia serpentina*	229	X	X	X

## 3 Data Description

We have collected 3494 images having 640 × 480 pixel dimensions and 96 × 96 dpi image resolution from the National Botanical Garden of Bangladesh and the Government Unani and Ayurvedic Medical College and Hospital which are located in Dhaka, Bangladesh. There are 16 different unique medicinal plant species. Collecting medicinal plants is an old tradition where plants are gathered from their natural homes or grown to use their healing benefits. These plants that have healing qualities have been used in both ancient and modern medicine. This dataset contains leaf images of sixteen rare, endangered and threatened medicinal plant species as described in Table 2.

**Table 2:** Medicinal plant leaf image classes and threat status.

SL	Classes	Local Name	Total images	Threat status
1	*Alternanthera brasiliana* L. *kuntze*	Kalochitra	201	Threatened
2	*Hemidesmus indicus (Linn.) R. Br.*	Anantamul	158	Rare [7]
3	*Achyranthes aspera*	Apang	280	Threatened
4	*Ayapana triplinervis*	Ayapan	294	Threatened
5	*Gloriosa superba*	Agnishikha	138	Rare [21]
6	*Andrographis paniculata wall. ex nees*	Kalomegh	294	Endangered [17]
7	*Datura tramonium* L.	Kalodhutura	276	Rare [26]
8	*Gynura procumbens (Lour.) merr.*	Gainura	238	Rare [1]
9	*Artocarpus chama*	Chapalish	238	Rare [9]
10	*Eleutherine bulbosa*	Betal	194	Threatened
11	*Boerhavia diffusa* L.	Punarnava	407	Rare [27]
12	*Clerodendrum indicum* L. *kuntze*	Bamonhati	134	Threatened
13	*Justicia adhatoda* L.	Basok	151	Endangered [10]
14	*Ocimum gatissimum* L.	Ram-Tulsi	154	Threatened
15	*Asparagus racemosus willd*	Shotomuli	108	Endangered [13]
16	*Rauwolfia serpentina*	Sarpagandha	229	Endangered [28]

Following are the 16 different classes and their description:

1. ***Alternanthera brasiliana* L. *Kuntze*:***Alternanthera brasiliana* L. *Kuntze*, usually known as ‘Kalochitra’ in Bengali is widely used in ayurvedic medicine. *Alternanthera brasiliana* L. *Kuntze*, the family of this plant is Amaranthaceae [23]. This is an herbaceous plant and a neotropical native species [23]. The entire *Alternanthera brasiliana* L. *Kuntze* plant has antibacterial activity [6]. It is an ornamental plant and has healing properties [23]. There are 201 total images in this category.
2. ***Hemidesmus indicus (Linn.) R. Br*:***Hemidesmus indicus,* usually known as ‘Anantamul’ in Bengali is being widely used in ayurvedic medicine [18]. The main constituents of the root are coumarin and volatile oil [18]. It also contains sterol, terpene, alcohol, lupeol, saponin, and tannin [18]. This is a slender and creeping type of plant. There are 158 original images in this category.
3. ***Achyranthes aspera*:***Achyranthes aspera* is a plant that is commonly called ‘Apang’ in Bengali. These plants that are used in traditional medicine have lots of different substances in them that can help with diseases that last a long time and with diseases caused by germs. These leaves contain tannins, flavonoids, glycosides, and alkaloids [3,23]. Apang is a perennial herbaceous plant. In this class, there are 280 total images in this category.
4. ***Ayapana triplinervis*:***Ayapana triplinervis* is a type of plant that many people in Bengal call ‘Ayapan’. Ayapan is a small bushy plant. An essential oil of A.triplinervis, where an important compound thymohy-droquinone dimethyl is found [24]. The whole Ayapan tree and the sap of the tree are used to make medicine [24]. There are 294 images in this group altogether.
5. ***Gloriosa superba*:***Gloriosa superba*, usually known as ‘Agnishikha’ in Bengali. It is a type of creeping plant, the leaves of which climb up with the help of pointed axils. It is a plant with lots of alka-loids, colchicine,superbine, gloriosine, lumicolchicine, 3-demethyl-N-deformyl-N-deacetylcolchicine, N-formyl deacetylcolchicine has been used for a long time as a traditional medicine in many different cultures [14]. But Agnishikha plants are deadly poisonous plants [16]. So its use and consumption are strictly forbidden without the permission of the expert. There are 138 total images in this category.
6. ***Andrographis paniculata Wall. Ex Nees*:***Andrographis paniculata* is a plant that is often called ‘Kalomegh’ in Bengali and is commonly used in ayurvedic medicine [12]. This plant has lots of natural chemicals like calomeghin and andrographolide, as well as other substances called lactones, diterpenes, flavonoids, quinic acid, xanthones, noriridoids, and other compounds [12]. It is a perennial plant. It is a herbaceous plant. There are 294 total images in this category.
7. ***Datura stramonium* L.:***Datura stramonium* L. is a plant that is commonly known as ‘Kalodhutura’ in Bengali. It has been used in folk remedies and alternative treatments for a long time. All parts of the Dhutura plant are poisonous and have medicinal properties [22]. The poisons present in the plant are tropane alkaloids [22]. However, if it is processed correctly, the plant can also have powerful medicinal properties [22]. There are a total of 276 images here.
8. ***Gynura procumbens (Lour.) Merr.*:***Gynura procumbens (Lour.) Merr.* is a plant that is often called ‘Gainura’ in Bengal. The English name is Longevity Spinach or Leaves of Life [30]. Gainura is a simple branched tree of the Virat class. Gainura is a wonderful plant, whose medicinal value is immense [30]. It also contains alkaloids, coumarins, flavonoids, triterpenes, and valepotriates [30]. That is why it is called the herb of longevity. In this class, there are 238 total images.
9. ***Artocarpus chama*:***Artocarpus chama,* usually known as ‘Chapalish’ in Bengali. This plant's fruit has Phenolic content and antioxidant activity which helps to reduce the risk of many diseases [11]. Bark, leaf, seed, and root are used for diarrhea, skin diseases, asthma, ulcers, etc. There are 238 original images in this category.
10. ***Eleutherine bulbosa*:** People have been using natural products from plants that have healing properties for a very long time to cure illnesses [20]. One of them is a plant called *Eleutherine bulbosa. Eleutherine bulbosa* is a type of plant with a bulb and it grows a bunch of leaves that look like grass. The plant is often taken from nature to be used locally as a medicine [20]. *E. bulbosa* has a fat, round root. This plant is an antibacterial, prebiotic, and antioxidant [20]. This category contains 194 images.
11. ***Boerhavia diffusa* L.:***Boerhavia diffusa* L. *Willd* is a plant that is often called ‘Punarnava’ in Ben- gal. Punarnava is a perennial creeping herb. Leaves and branches contain triterpenoids, lipids, lignins, carbohydrates, proteins, glycoproteins, alkaloids, potassium salts, and saponin glycosides in the root [15]. In this category, there are 407 total images.
12. ***Clerodendrum indicum* L. *Kuntze*:***Clerodendrum indicum* is a type of plant that many people in Bengal call ‘Bamonhati’. The plant's different parts like root, bark, flower, and leaf contain active constituents of phenolic glycosides and saponins [19]. Bamanhati or Bonchat is a branched perennial shrub with a hollow stem [19]. There are 134 images in this group.
13. ***Justicia adhatoda* L.:***Justicia adhatoda* L. is a type of plant that many people in Bengal call ‘Basok’. It is a herb whose leaves, roots, flowers, and other parts are used in medicine [3]. Basak is used in many types of medicine, such as Herbal, Ayurvedic, and Unani. Basak looks like a thick bush [3]. In this class, there are 151 images.
14. ***Ocimum gratissimum* L.:***Ocimum gratissimum* L. is a plant that is commonly called ‘Ram-Tulsi’ in Bengali. Ram-Tulsi is also used in herbal preparations in many Ayurvedic and naturopathic hospitals due to its medicinal properties [29]. Tulsi leaves of this variety have a sweet taste. The substances that have health benefits that are taken out from O.Gratissimum are substances called phytochemicals, which are oleanolic acid, caffeic acid, ellagic acid, epicatechin, sinapic acid, rosmarinic acid, chlorogenic acid, luteolin, apigenin, nepetoidin, xanthomicrol, neva-densin, salvigenin, gallic acid, catechin, quercetin, rutin, and kaempferol, and also has essential oils [29]. There are 154 total images in this category.
15. ***Asparagus racemosus Willd:****Asparagus racemosus Willd* is a plant that is often called ‘Shotomuli’ in Bengal. The herbal properties of ‘Shotomuli’ are immense. Its name is Shotomuli because it has knotted clusters of roots like garlands. Satamuli contains high-quality folic acid and is an excellent natural source of potassium, e.g., Mg, P, Ca, Fe [8]. It also contains fiber, vitamin A, vitamin B1, B2, and other compounds in roots [8]. This is a perennial creeping plant. There are 108 total images in this class.
16. ***Rauwolfia serpentina*:***Rauwolfia serpentina* is a plant that is commonly known as ‘Sarpagandha’ in Bengal [5]. Sarpagandha root contains indole alkaloids including reserpine, diserpine, and resinamine [25]. Others include ajmaline, ajmalysin, serpentine, oleoresin, and unsaturated alcohols [25]. A herbaceous plant that grows in the form of an upward bush. In this set of information, there are a total of 229 images.

We collected the data throughout August 2023, capturing images in various weather conditions (sunny, rainy, cloudy) and at different times of the day (morning, noon, afternoon). Multiple cameras were used, but we ensured a consistent resolution across all images. The plants were primarily located at The Government Unani and Ayurvedic Medical College and Hospital and The National Botanical Garden of Bangladesh. Our goal was to observe any differences in images due to weather and daylight variations. Since after collecting the leaves, we captured all the images under shaded/indoor areas, no significant changes were found in the images.

Table 3 provides the details of the photo collection of our dataset, including the local name of each plant, the weather conditions during which photo were captured, the date, the devices used for photography, and the specific locations where the photos were taken. This detailed information could be utilized for a better understanding of the dataset and its relevance to the study.

**Table 3:** Medicinal plant leaf images data collection details.

Local plant name	Weather	Date	Time	Camera Devices	Location
Kalomegh, Shotomuli	Rainy	18 August 2023	Noon	Samsung Galaxy S21+ 5G (100 %)	The National Botanical Garden of Bangladesh, Mirpur, Dhaka 1216.
Apang	Cloudy	18 August 2023	Noon	Redmi Note 11 (50 %) and Samsung Galaxy S21+ 5G (50 %)	The National Botanical Garden of Bangladesh, Mirpur, Dhaka 1216.
Kalochitra, Kalodhutura, Basok, Sarpogondha	Sunny	18 August 2023	Afternoon	Samsung Galaxy S21+ 5G (100 %)	The National Botanical Garden of Bangladesh, Mirpur, Dhaka 1216.
Agnishikha	Sunny	19 August 2023	Afternoon	Redmi Note 11 (100 %)	The National Botanical Garden of Bangladesh, Mirpur, Dhaka 1216.
Gainura, Punarnava, Kalomegh, Anantamul, Apang, Kalodhutura, Ayapan, Sarpagandha	Sunny	25 August 2023	Noon	Samsung Galaxy S21+ 5G (100 %)	The Government Unani and Ayurvedic Medical College and Hospital, Mirpur Bridge Road, Dhaka 1216.
Bamonhati	Sunny	25 August 2023	Afternoon	Redmi Note 11 (100 %)	The Government Unani and Ayurvedic Medical College and Hospital, Mirpur Bridge Road, Dhaka 1216.
Chapalish, Betal	Sunny	26 August 2023	Morning	Samsung Galaxy S21+ 5G (100 %)	The National Botanical Garden of Bangladesh, Mirpur, Dhaka 1216.

**Fig. 1:**
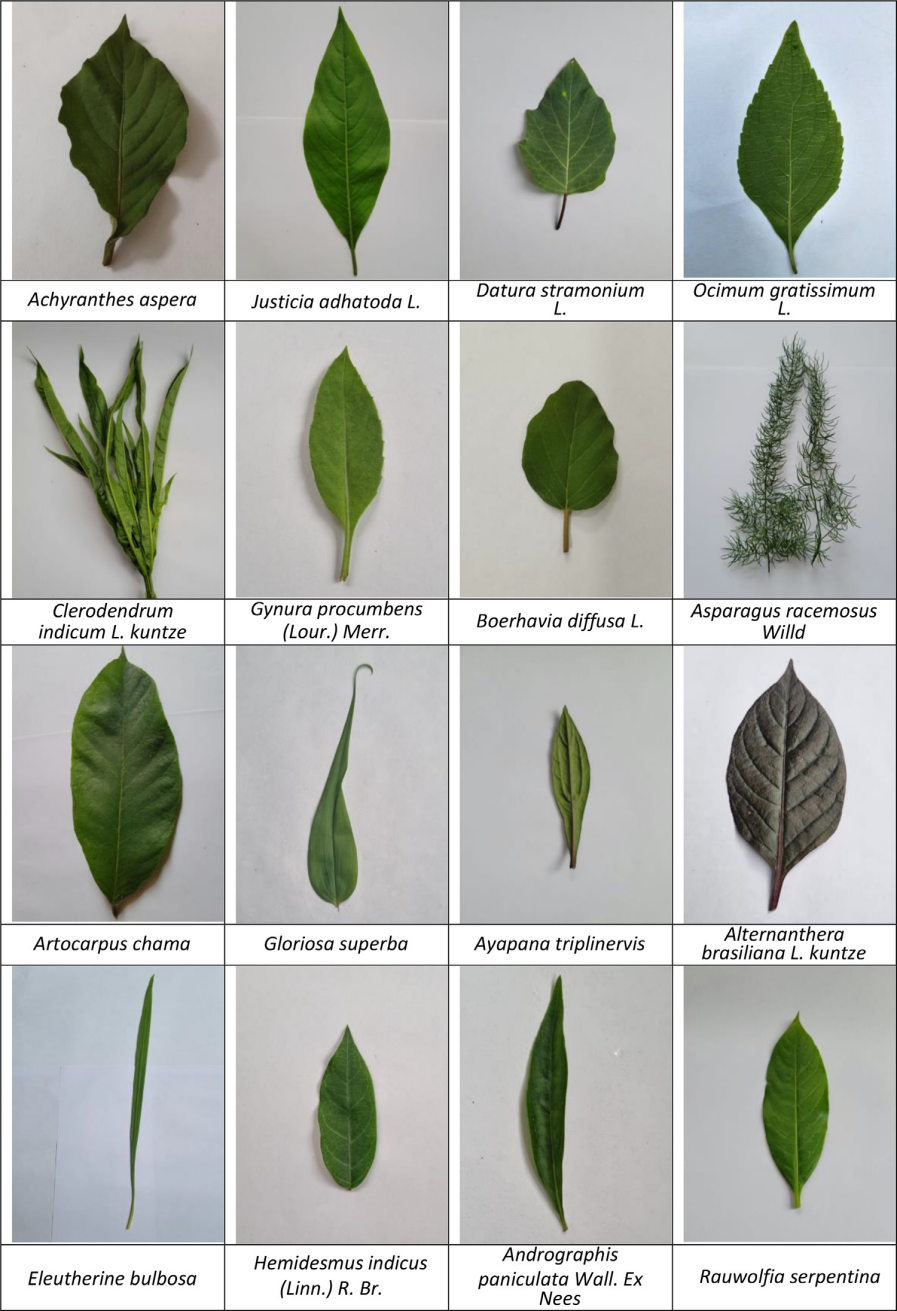
Sample of 16 different types of medicinal leaf image sample from image dataset.

Fig. 1 below displays sample image from each class in the dataset, providing an overview of the diversity within the dataset.

## 4 Experimental Design, Materials and Methods

The medicinal plant images of healthy leaves were acquired using smartphone cameras at the same working distance without zoom. We have collected 3494 images of plant leaves throughout this data-collecting procedure. We used natural light and a white background to capture the images. We also captured the images of plant leaves in different weather. We also collected leaves from different locations. Further scientific investigation and clinical experimentation are crucial to fully understand and utilize the healing abilities of plants with medicinal qualities. They test and confirm that traditional knowledge about herbal remedies is accurate and that these remedies are both safe and effective. This research could help create new drugs to treat different health problems. Furthermore, it helps with gathering resources in a way that doesn't harm the environment or deplete them completely, making sure that future generations can still benefit from these valuable resources.

For machine learning models, a high-quality dataset is crucial since it provides the basis for efficient training and generalization. By offering a realistic portrayal of many situations, it guarantees that the model gains knowledge from a wide variety of instances. The dataset's balance and lack of biased representations aid in preventing biases. Adequate feature coverage is essential for obtaining pertinent data, while a big enough dataset keeps the model from overfitting and enhances its generalization capabilities.

## 5 Methodology

**Fig. 2:**
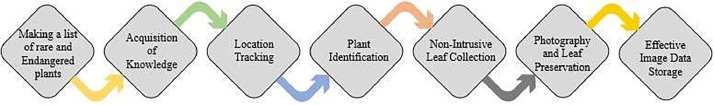
Process steps of the whole workflow.

The essence of successful plant identification lies in having a robust dataset. Quality datasets, in terms of size, intra-class integrity, inter-class dissimilarity, and absence of noise, are paramount for accurate predictions. For the specific goal of identifying rare medicinal plants, the development of a standardized, ready-to-use, and publicly available dataset of images becomes pivotal. This dataset aims to represent the real-life scenario where the identification system will be applied, ensuring the relevance and applicability of the technology to diverse settings. The diagram shows the steps of each process below (Fig. 2):

1. *Making list of rare and endangered plants:* Begin by creating a comprehensive list of rare and endangered medicinal plants. This involves thorough research and consultation with botanical databases to ensure a comprehensive compilation.
2. *Acquisition of Knowledge:* Gather in-depth knowledge about each identified medicinal plant, including its habitat, growth conditions, and unique features. Consult botanical experts, literature, and reliable online resources to enhance understanding.
3. *Location Tracking:* Utilize geographical information systems (GIS) and botanical surveys to pinpoint possible locations of the identified rare and endangered medicinal plants. Collaborate with local experts and communities to gather insights on plant habitats.
4. *Plant Identification:* Implement botanical identification techniques to accurately identify the rare and endangered medicinal plants in the field. Utilize field guides and expert assistance to ensure precision.
5. *Non-Intrusive Leaf Collection:* Collect plant leaves with the utmost care, emphasizing non- intrusive methods to ensure minimal harm to the plants. Employ ethical harvesting practices and prioritize the preservation of the plant's natural environment.
6. *Photography and Leaf Preservation:* Capture high-quality photographs of the identified plants, focusing on key characteristics for future reference. Simultaneously, carefully preserve collected leaves using appropriate methods to maintain their botanical integrity.
7. *Effective Image Data Storage:* Establish a systematic approach to storing image data, ensuring proper organization and accessibility. Implement a secure and well-structured database to store photographs and associated information for future research and conservation efforts.

## Limitations

It was challenging to gather a lot of data for some plants as they were not widely available. For this reason, we could not collect a huge number of leaves from rare and endangered plants. Consequently, certain classes have a lower number of leaf images. However, researchers can apply many augmentation techniques and increase the number of samples as required for their machine learning model training. Additionally, the six ‘threatened’ classes were identified through name tag and description written at the data collection spot; we were unable to find research papers regarding the six threatened medicinal plants in Bangladesh.

## Ethics Statement

The study was conducted strictly in compliance with ethical guidelines, indicating a dedication to the highest standards. No plants or animals were harmed in any way throughout the data-collecting procedure. It is significant to remember that every image was taken with the explicit approval of the owners, including those in charge of nurseries, gardens, plants, and other such establishments. It only includes the images that were obtained by the author and the current work does not involve human subjects, animal experiments, or any data collected from social media platforms.

## CRediT Author Statement

**Mohammad Manzurul Islam:** Supervision, Conceptualization, Project Administration, Investigation, Writing – review & editing; **Sanjida Rahman:** Data curation; Writing – original draft, Validation; **Nahida Hoque:** Data curation; Writing - original draft, Visualization; **Md. Al Mamun:** Data curation; Conceptualization; **Md. Sultan Moheuddin:** Writing – original draft, Methodology; **Md. Sawkat Ali:** Visualization, Validation; **Mohammad Rifat Ahmmad Rashid:** Supervision; **Saleh Masum:** Validation; **Md. Hasanul Ferdaus:** Investigation, Visualization; **Md. Atiqur Rahman:** Project Administration.

## Data Availability

REMP: A Unique Dataset of Rare and Endangered Medicinal Plants in Bangladesh (Original data) (Mendeley Data).
